# Dysregulation of miR-21-associated miRNA regulatory networks by chronic ethanol consumption impairs liver regeneration

**DOI:** 10.1152/physiolgenomics.00113.2021

**Published:** 2021-11-19

**Authors:** Austin Parrish, Ankita Srivastava, Egle Juskeviciute, Jan B. Hoek, Rajanikanth Vadigepalli

**Affiliations:** Daniel Baugh Institute for Functional Genomics and Computational Biology, Department of Pathology, Anatomy and Cell Biology, Thomas Jefferson University, Philadelphia, Pennsylvania

**Keywords:** alcoholic liver disease, hepatic stellate cells, liver regeneration, microRNA networks

## Abstract

Impaired liver regeneration has been considered as a hallmark of progression of alcohol-associated liver disease. Our previous studies demonstrated that in vivo inhibition of the microRNA (miRNA) miR21 can restore regenerative capacity of the liver in chronic ethanol-fed animals. The present study focuses on the role of microRNA regulatory networks that are likely to mediate the miR-21 action. Rats were chronically fed an ethanol-enriched diet along with pair-fed control animals and treated with AM21 (anti-miR-21), a locked nucleic acid antisense to miR-21. Partial hepatectomy (PHx) was performed and miRNA expression profiling over the course of liver regeneration was assessed. Our results showed dynamic expression changes in several miRNAs after PHx, notably with altered miRNA expression profiles between ethanol and control groups. We found that in vivo inhibition of miR-21 led to correlated differential expression of miR-340-5p and anticorrelated expression of miR-365, let-7a, miR-1224, and miR-146a across all sample groups after PHx. Gene set enrichment analysis identified a miRNA signature significantly associated with hepatic stellate cell activation within whole liver tissue data. We hypothesized that at least part of the PHx-induced miRNA network changes responsive to miR-21 inhibition is localized to hepatic stellate cells. We validated this hypothesis using AM21 and TGF-β treatments in LX-2 human hepatic stellate cells in culture and measured expression levels of select miRNAs by quantitative RT-PCR. Based on the in vivo and in vitro results, we propose a hepatic stellate cell miRNA regulatory network as contributing to the restoration of liver regenerative capacity by miR-21 inhibition.

## INTRODUCTION

Alcohol-associated liver disease (ALD) is a major health concern and one of the leading causes of chronic liver disease, eventually leading to the development of cirrhosis and hepatocellular carcinoma (1, 2). Although the liver is normally capable of restoring its baseline mass and function following severe injury, the regenerative capacity of the liver is significantly decreased by chronic ethanol consumption, due to complications arising from hepatitis, fibrosis, and improper hepatocyte proliferation (2). Transcriptomic approaches to understanding these changes in liver physiology have revealed reprogramming of multiple molecular systems, suggesting the necessity of a systemic approach to better understand alcohol-associated liver disease (3, 4). Of particular interest are the observed changes in hepatic stellate cell (HSC) signaling networks, which can lead to the transition between multiple functional cell states such as quiescent (baseline) and activated (antiproliferative, profibrotic). Regulation of the signaling resulting in shifts to these states, and thus overall HSC function, has significant implications for the regenerative capacity in the liver. Recent studies have shown microRNA (miRNA) to be a particularly important regulatory mechanism for the control of cell function and may play a significant role in the transition between cell states. miRNAs are a class of small, noncoding RNAs ∼22 nucleotides in length that are involved in post-transcriptional regulation of a wide variety of processes critical for normal cell function, such as development, cell differentiation, cell proliferation, and cell death (5–7). Previous research has investigated the critical role of miRNA dysregulation in multiple disease states across organ systems, including liver-specific diseases such as infection with hepatitis B or C viruses (HBV/HCV) and hepatocellular carcinoma (HCC) (7). However, the majority of these studies focused only on a small number of miRNAs and offered little insight into the dynamics of their expression levels. There remains, therefore, a dearth of knowledge regarding the precise role and function of miRNAs in the context of ALD. Previous work by our laboratory and others has identified several miRNAs to be key regulators of liver regeneration, such as miR-21, whose role in liver regeneration and liver disease has been well studied both in vivo and in vitro with a significant role in HSC activation via the TGF-β signaling pathway (8–12). We have previously shown that chronic ethanol (EtOH) consumption in rats leads to an elevated expression of miR-21 in post-partial hepatectomy (PHx) relative to pair-fed control animals. To our knowledge, complex expression dynamics of miRNA during liver regeneration has not been examined in depth. In this study, we sought to characterize global miRNA expression patterns in the rat liver for both control and ethanol-fed animals before and after 70% PHx, including nonparenchymal cell signatures.

## MATERIALS AND METHODS

### Animal Model

All animal experiments were carried out in accordance with protocols approved by the Thomas Jefferson University Institutional Animal Care and Use Committee. Male Sprague–Dawley rats (Charles River, Wilmington, MA), between the ages of 8 and 14 wk were pair-fed an isocaloric liquid diet according to the Lieber–DeCarli protocol (13) for 6–8 wk, in which 36% of total calories came either from ethanol (EtOH) or from carbohydrates (CHO; maltose-dextran) in control animals (Bio-Serv, Frenchtown, NJ). Animals were maintained on a 12:12-h light-dark cycle. Liver damage was induced by a 70% partial hepatectomy (PHx) surgery in which the left lateral and medial lobes were removed following anesthetization with isoflurane. Livers were allowed to regenerate for 6, 24, or 72 h before the remaining liver was excised. Total RNA from each sample was extracted from frozen liver samples using an Animal Tissue RNA Purification Kit (Norgen Biotek, Thorold, ON, Canada).

### In Vivo Inhibition of miR-21

Rats were injected with 7.5 mg/kg ip of locked nucleic acid (LNA) oligonucleotide probe antisense to miR-21 (Exiqon, Vedbaek, Denmark) in 1 mL of saline. These anti-miR-21 (AM21) injections were given at two time points—one 72 h before surgery and one immediately following PHx surgery. Control animals were either left untreated or injected intraperitoneally with 1 mL of saline.

### Cell Culture and Treatments

The LX-2 human HSC line was obtained from Millipore Sigma (Burlington, MA). LX-2 cells were cultured in Dulbecco’s modified Eagle’s media (DMEM) (Gibco, Waltham, MA) supplemented with 1% fetal bovine serum (FBS) (Gibco) and 100 U/mL penicillin and 100 U/mL of streptomycin (Gibco) at 37°C, 5% CO_2_. LX-2 cells were seeded at 3.5 × 10^5^ cells/well in a six-well culture plate. The cells were starved and transfected at 80% cell confluence with one of anti-miR hsa-miR-21-5p miRCURY LNA miRNA Power Inhibitor 5′ FAM-labeled (Qiagen, Hilden, Germany). All treatments were performed using 50 nM LNA for 72 h following the manufacturer’s protocol. Cell stimulation with TGF-β (Peprotech, Rocky Hill, NJ) was carried out at 5 ng/mL for 48 h. The cells were harvested using Qiazol (Qiagen) for RNA isolation.

### NanoString nCounter Array

Purified RNA from rat livers was assayed using NanoString’s in-house services against a list of 420 well-characterized rat miRNAs. Raw counts were normalized first by Trimmed Mean of M values (TMM) normalization using the “edgeR” package version 3.34.1 as developed for the R programming language (14), followed by voom transformation (15) using the “limma” package version 3.48.3 (16) to convert counts to log-cpm. Differential expression analysis was performed using “limma” with log-cpm values (17). Data are available at Gene Expression Omnibus (GEO) accession ID GSE171438. Pair-wise Pearson correlation coefficients between miRNAs were calculated based on the normalized expression data using the *cor* function available in the base package of the R software version 3.6.

### RNA Isolation and Real-Time Quantitative PCR Analysis

For quantitative real-time PCR analysis, RNA was isolated using miRNeasy Mini Kit (Qiagen). For quantification of miRNA expression, 10 ng of total RNA was used, and cDNA was synthesized using TaqMan Advanced miRNA cDNA Synthesis Kit (Thermo Fisher, Waltham, MA). Real-time PCR analysis was performed to measure the miRNA expression levels using TaqMan Fast Advanced Master Mix and Taqman primers with conditions (enzyme activation: 95°C for 20 s; 40 PCR cycles of denaturation: 95°C for 1 s; annealing and extension: 60°C for 20 s). Target micro-RNA expression was normalized using three endogenous miRNAs (hsa-miR-99b-5p, hsa-miR-23a-3p, and hsa-miR-100-5p). Relative expression of miRNA was analyzed using the ΔΔcomparative threshold (CT) method. The statistical significance was performed using GraphPad Prism unpaired *t* test, and values are means ± SE (*n* = 4), ****P < 0.0001, ***P < 0.001, and **P < 0.01.

### Immunofluorescence Staining

LX-2 cells were cultured on eight-well chamber slides at a confluence of 10 × 10^3^ cells. After LNA transfection and TGF-β stimulation, the cells were fixed in 4% paraformaldehyde (Electron Microscopy Sciences, Hatfield, PA) for 20 min and washed with 1× PBS (Fisher Bioreagents), two washes for 5 min each. The cells were blocked using 5% normal goat serum (ab7481; Abcam, Cambridge, MA) for 1 h. All steps including fixation and blocking were done at room temperature. Blocking was followed by overnight incubation at 4°C followed by three washes of 1× PBS at 5-min intervals each. The primary antibody targeted alpha smooth muscle actin (αSMA) (M085101, Agilent, Santa Clara, CA) used at 1:50 dilution. The antibody was chosen based on previously published literature regarding hepatic stellate cell activation comparing primary HSCs isolated from male Wistar rats cultured over 7 days; quiescent cells showed no α smooth muscle actin expression, whereas activated cells showed high levels (18). The secondary antibody was used at 1:500 dilutions, goat anti-mouse IgG Alexa Fluor 488 (ab150117, Abcam). The fixed cells were incubated with secondary antibodies for 1 h 45 min at room temperature followed by three washes of 5 min each with 1× PBS. After secondary antibody incubation, DAPI (D9542; Sigma-Aldrich) was applied and allowed to incubate for 15 min followed by three washes of 5 min each. ProLong Diamond Antifade (Life Technologies) solution was applied, and the slides were mounted with coverslips. Prior to microscopic imaging, slides were cured at room temperature overnight. Images were acquired using a Zeiss LSM 780 mounted on a Zeiss Axio Observer inverted microscope. Images were acquired using a Zeiss LSM 780 mounted on a Zeiss Axio Observer inverted microscope. Zeiss ZEN 2011 software package associated with the LSM 780 was used to set the image acquisition parameters and capture images. Laser emission at wavelengths of 405 nm and 488 nm were used for image acquisition to capture DAPI and the secondary antibody against α smooth muscle actin, respectively. Prior to the image acquisition, the range of the accepted signal levels (i.e., zero and saturation parameters) were set using the range indicator function built into the Zeiss software. Images were acquired at a pixel resolution of 1,024 × 1,024 at 8-bit color depth with a line scan and averaging intensities from four scans of the same area.

### miRNA Target Identification

Putative miRNA target genes were retrieved from target site predictions using output from the miRanda algorithm (August 2010 release) (19). Predicted targets were chosen with high scores and conserved miRNA families. Networks of predicted targets were constructed using Cytoscape v3.2.1. Gene ontology analysis was performed using DAVID (https://david.ncifcrf.gov/; version 6.7) (20).

### Gene Set Enrichment Analysis

Gene set enrichment analysis (GSEA) was performed using a set of R scripts developed by the Broad Institute. Briefly, miRNA expressed in control or alcohol-fed animal samples were tested against a known set of miRNAs associated with HSCs, rat liver sinusoidal endothelial cells (LSECs), or Kupffer cells (KCs) to determine if these miRNA sets display significant correlation with expression patterns from a particular biological state. These miRNA signatures were derived from previously published miRNA profiling data from primary rat hepatic stellate cells activated in culture (21), rat liver sinusoidal endothelial cells (LSECs) compared with other liver cell types (22), and mouse Kupffer cells (KCs) at 3 days after 70% PHx (23). The HSC signature consisted of 16 upregulated miRNAs and 26 downregulated miRNAs. The LSEC signature consisted of 66 miRNAs enriched in LSECs compared with other liver cell types [highlighted in Figure 2A of Oda et al. (22)]. The KC signature consisted of 20 differentially expressed miRNAs. This set was derived by analyzing the normalized miRNA expression data available via Gene Expression Omnibus data set GSE159198, using *limma* function in R to compare the 3-day post PHx versus 0-h samples, and filtered for false discovery rate adjusted *P* value <0.1 and top 20 log2(fold change) values.

## RESULTS

### Multiple miRNAs Are Differentially Expressed in Response to Ethanol Adaptation and Further Altered by PHx

**Figure 1.**
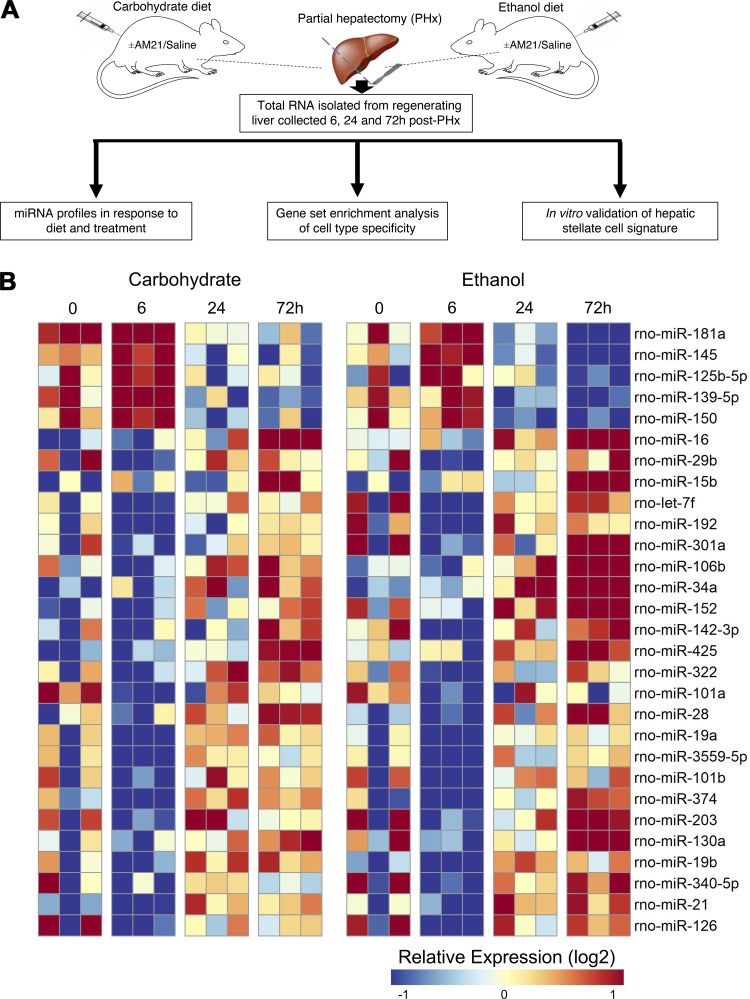
*A*: overview of experimental model of liver regeneration and subsequent analysis. Sprague-Dawley rats were fed a liquid diet containing either ethanol or a carbohydrate mixture. Liver regeneration was induced by a 70% partial hepatectomy and allowed to regenerate up to 72 h. Animals treated with anti-miR-21 (AM21) or saline were injected intraperitoneally 72 h prior to surgery and immediately thereafter; samples were collected at baseline and 24 h after partial hepatectomy (PHx). Total RNA from rat liver was extracted for a NanoString miRNA assay and further validated in vitro. *B*: heatmap of differentially expressed miRNAs from untreated animals. miRNAs show both time- and diet-dependent changes in expression. *n* = 3 animals per condition.

**Figure 2.**
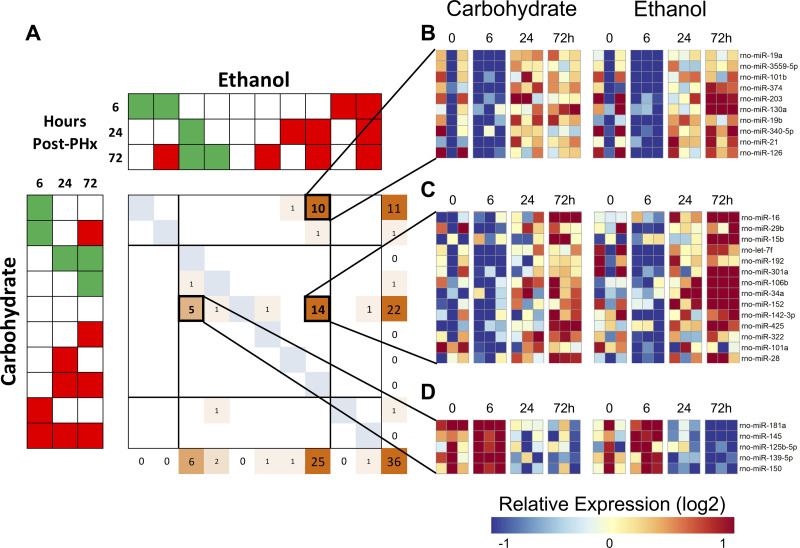
Comparative pattern count (COMPACT) analysis reveals clusters of similarly altered miRNAs. *A*: overview of the COMPACT matrix showing discrete groups of miRNAs arranged in accordance with changes in expression between carbohydrate- and ethanol-fed animals across time points. These groups (*B*, *C*, and *D*) show that multiple miRNAs display altered expression levels at early, intermediate, and later time points of regeneration. PHx, partial hepatectomy.

Male Sprague-Dawley rats were fed a liquid diet according to the Lieber–DeCarli protocol (13). Liver regeneration was induced by 70% partial hepatectomy and the regenerating liver tissue was collected at 6, 24, and 72 h after PHx (Fig. 1*A*). Total RNA was extracted from whole liver rat tissue for NanoString analysis of 420 miRNAs. During recovery following 70% PHx, 36 miRNAs were differentially expressed by 72 h in both groups (Fig. 1*B*). We next sought to quantitatively compare the differences in expression profiles between diets across time. To this end, we employed a method developed by our laboratory [comparative pattern count (COMPACT) analysis] (10). Briefly, this approach uses discretized changes compared with a baseline sample (e.g., 24 h vs. 0 h post PHx). Discretized patterns are encoded from a predetermined fold change threshold to classify time points or conditions as upregulated, downregulated, or unchanged. Differentially expressed miRNAs are assigned to groups with distinct expression patterns, with each group containing unique sets of miRNAs (Fig. 2). From this analysis, three sets of miRNAs were immediately noticeable—those downregulated at 6 h in CHO-fed animals but upregulated at 24 and 72 h in EtOH-fed animals (Fig. 2*B*), those upregulated by 24 and 72 h in the EtOH-fed animals only (Fig. 2*C*), and those downregulated at 24 and 72 h in EtOH-fed animals but unchanged in CHO (Fig. 2*D*). Six miRNAs are significantly downregulated at later time points in ethanol-adapted livers and 25 miRNAs are significantly upregulated; 10 of these miRNAs show a decrease at 6 h in carbohydrate-fed animals compared with ethanol-fed, while the remaining 14 show no significant changes in carbohydrate-fed animals (Fig. 2; significance determined by log2 fold change ≥ 1.5). One of the miRNAs to show significant upregulation in ethanol-fed animals, miR-21, has been previously observed as an important regulator of liver regeneration (12, 24).

### Inhibition of miR-21 In Vivo Alters the Expression Level of Several Additional miRNAs

**Figure 3.**
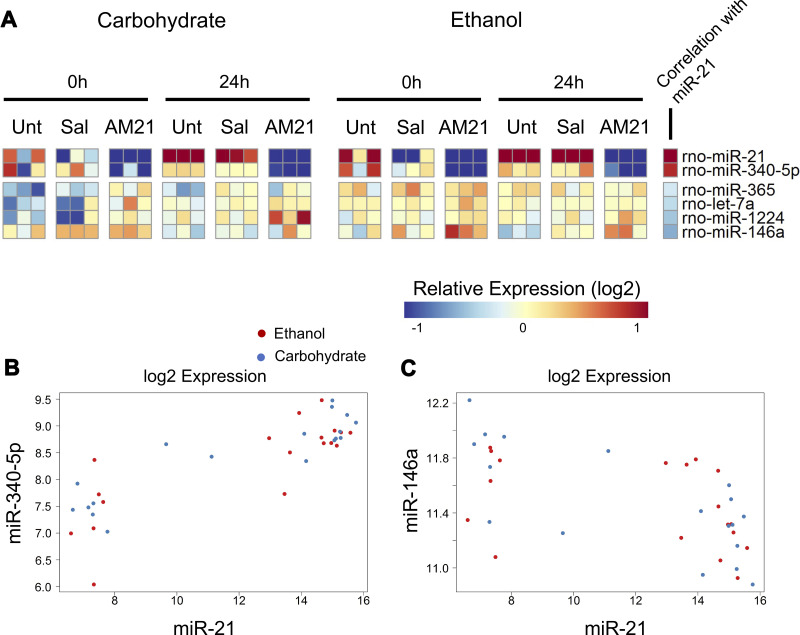
*A*: dynamics of differentially expressed miRNAs in response to anti-miR-21 (AM21) treatment, along with Pearson correlation to miR-21 (red = positively correlated, blue = negatively correlated). *n* = 3 biological replicates for all groups. Log2-transformed expression values for the most positively correlated (*B*) and most negatively correlated (*C*) miRNAs.

Previous study from our group has shown that inhibition of miR-21 in vivo rescues the delayed liver regeneration phenotype observed in ethanol-fed rats post PHx and leads to broad changes in gene expression (9). Based on the spectrum of molecular changes observed in this previous study, we hypothesized that miR-21 is involved in a broader gene regulation network affecting other co-regulatory miRNAs. We treated carbohydrate-fed (CHO) and ethanol-fed (EtOH) animals with either anti-miR-21 injections (AM-21), a locked nucleic acid (LNA) oligonucleotide complementary to miR-21, saline injections, or left untreated. PHx surgery was performed within each group and animals were allowed to recover for 24 h post PHx before remnant liver tissue was collected. Expression level of one miRNA from treated rats (miR-3405p) showed significant positive correlation to miR-21 (Pearson *r* > 0; *P* < 0.05; Fig. 3, *A* and *B*), whereas four miRNAs (miR-365, let-7a, miR-1224, and miR-146a) showed significant negative correlation (Pearson *r* < 0; *P* < 0.05) (Fig. 3, *A* and *C*). miR-146a, which was most anticorrelated with miR-21 expression (Fig. 3*C*), is reported to act as a tumor suppressor and is involved in the regulation of hepatic stellate cell activity, suggesting a possible functional role of this miRNA in controlling gene expression in the context of response to partial hepatectomy following miR-21 inhibition (21, 25–27).

We used the miRanda target prediction software to identify putative targets of the miRNAs responsive to AM-21 treatment. Enrichment analysis of the targeted genes was performed with the DAVID bioinformatics resource (version 6.7) (20). We identified several significantly enriched processes by which these miRNAs may regulate liver regeneration, mainly involved in cell cycle progression and cell death (Table 1). Taken together, these results suggest that multiple miRNAs are involved in co-regulatory networks, and that disruption of normal miRNA expression (e.g., by chronic ethanol consumption) leads to a cascade of changes in gene expression, contributing to dysregulated liver regeneration following injury.

**Table 1.** Table of gene ontology terms from DAVID (version 6.7) associated with predicted targets of miR-21, miR-340-5p, miR-365, miR-146a, and let-7a

GO Term	Gene Count	FDR
Transcription (GO:0006350)	147	2.56E−08
Regulation of programmed cell death (GO:0043067)	146	6.49E−08
Regulation of cell proliferation (GO:0042127)	138	1.47E−06
Response to hypoxia (GO:0001666)	54	1.67E−05
Response to wounding (GO:0009611)	95	1.94E−05
Positive regulation of developmental process (GO:0051094)	74	7.91E−05
Positive regulation of cell differentiation (GO:0045597)	59	0.003798

DAVID, Database for Annotation, Visualization and Integrated Discovery; FDR, false discovery rate.

### GSEA Uncovers a Signature for Hepatic Stellate Cell miRNAs in Liver Regeneration

**Figure 4.**
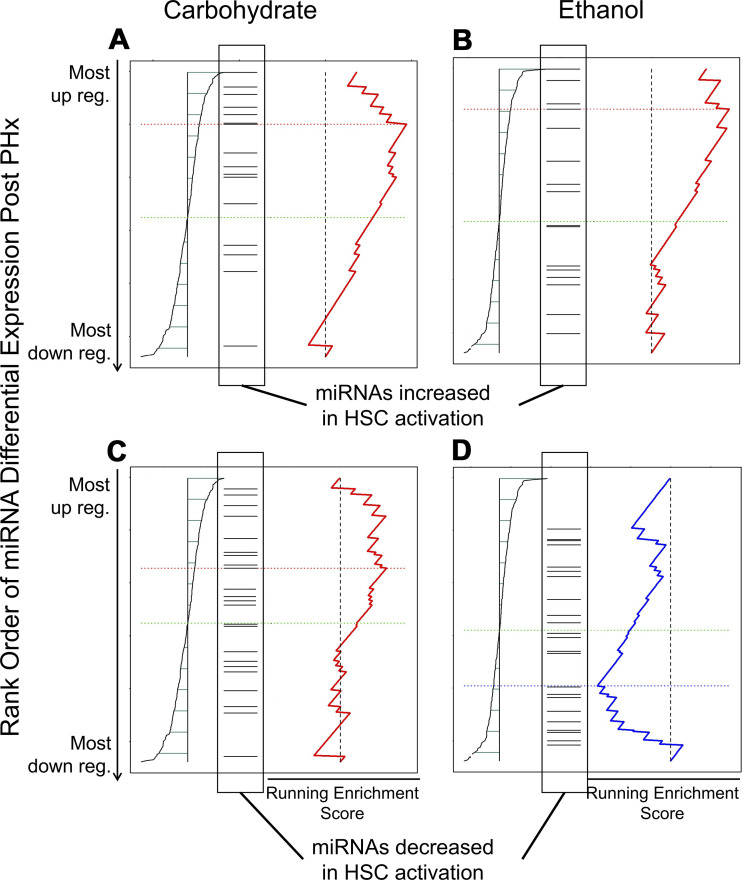
Gene set enrichment analysis reveals changes in miRNA expression patterns that reflect those seen in hepatic stellate cell activation (11). At 24-h post-partial hepatectomy (PHx), both control and alcohol-fed animals show an enrichment in miRNAs upregulated with stellate cell activation (*A* and *B*). Alcohol-fed animals prior to surgery show a slight enrichment for miRNAs downregulated with hepatic stellate cell activation (*D*), whereas control animals do not appear to show enrichment for these miRNAs in either condition (*C*).

To further investigate the potential link between dysregulation of normal miRNA expression in nonparenchymal cells and liver regeneration, we performed gene set enrichment analysis comparing miRNA differential expression in the ethanol and carbohydrate control groups to known sets of miRNAs that are differentially expressed in HSCs (21), LSECs (22), and KCs (23) (Table 2). The HSC-associated miRNA signature consisted of 16 upregulated and 26 downregulated miRNAs, of which all but 3 downregulated miRNAs overlapped with our data set. Our analysis revealed that by 24-h post-PHx there is significant enrichment in both ethanol-fed and control groups for miRNAs that are upregulated in activated HSCs (Fig. 4, *A* and *C*). The carbohydrate control group did not show significant enrichment for miRNAs that are downregulated in activated HSCs (Fig. 4*B*). However, ethanol group displayed a statistically significant enrichment for this set of downregulated miRNAs, showing decreased expression after PHx (Fig. 4*D*). For LSECs, we used a list of miRNAs enriched in these cells relative to other liver cell types and evaluated the over-representation of the LSEC-enriched miRNA signature in the bulk tissue miRNA profiling data from our experiments. Of the 66 miRNAs included in this signature, only 30 miRNAs overlapped with our bulk tissue data set (Table 2). This signature corresponding to the LSEC-enriched miRNAs was not statistically significant in the carbohydrate (*P* = 0.68) or ethanol (*P* = 0.28) groups. For assessing the potential contribution of KC-associated miRNAs, we analyzed a miRNA profiling data set collected from isolated KCs at 3 days after 70% PHx in mice to identify a differential miRNA expression signature to use in the GSEA (Table 2). Of the 20 miRNAs included in this signature, only 7 miRNAs overlapped with our bulk tissue data set. GSEA results indicated poor statistical significance of the KC signature in carbohydrate (*P* = 0.13) and ethanol (*P* = 0.69) groups. Taken together, these results suggest that it is difficult to ascertain the contribution of LSEC- and KC-associated miRNA networks from the present bulk tissue data set, even as HSC-associated miRNA signatures were prominently identifiable using this statistical approach.

**Table 2.** Table of nonparenchymal cell-associated miRNA signatures used in the gene set enrichment analysis

Hepatic Stellate Cells Upregulated	Hepatic Stellate Cells Downregulated		Liver Sinusoidal Endothelial Cells		Kupffer Cells	
Overlapping	Overlapping	Nonoverlapping	Overlapping	Nonoverlapping	Overlapping	Nonoverlapping
let-7b	miR-207	miR-10a	miR-351	miR-126a-3p	miR-483	miR-671-3p
let-7c	miR-335	miR-151*	miR-335	miR-126a-5p	miR-99b	miR-1901
let-7e	miR-195	miR-422b	miR-146b	miR-322-3p	miR-103	miR-465c-5p
miR-125b-5p	miR-16		miR-195	miR-511-3p	miR-539	miR-331-5p
miR-132	miR-30a		miR-322	miR-450b-5p	miR-96	miR-466c-5p
miR-143	miR-122		miR-374	miR-143-3p	miR-29b	miR-383
miR-145	miR-30d		miR-16	miR-23a-3p	miR-152	miR-m108-2-5p
miR-152	miR-30b-5p		miR-199a-5p	miR-338-3p		miR-883b-5p
miR-199a-5p	let-7f		miR-10b	miR-130a-3p		miR-467e
miR-21	miR-30c		miR-497	miR-27a-3p		miR-433
miR-210	miR-194		miR-140	miR-130b-3p		miR-742
miR-214	miR-192		let-7i	miR-362-3p		miR-193b
miR-22	miR-29a		miR-142-3p	miR-24-3p		miR-380-5p
miR-221	miR-26b		miR-466d	miR-23b-3p		
miR-222	miR-126		miR-532-5p	miR-29b-3p		
miR-31	miR-146a		miR-99b	miR-24-2-5p		
	miR-296		miR-151	miR-500-3p		
	miR-125a-5p		miR-142-5p	miR-381-3p		
	miR-26a		miR-146a	miR-301a-3p		
	miR-99a		miR-125a-5p	miR-3473		
	miR-181a		miR-145	miR-1247-3p		
	miR-150		miR-450a	miR-127-3p		
	miR-483		miR-10a-5p	miR-214-3p		
			miR-139-5p	miR-24-1-5p		
			miR-181a	miR-6318		
			miR-150	miR-211-3p		
			miR-328a	miR-494-3p		
			miR-532-3p	miR-501-3p		
			miR-362	miR-331-3p		
			miR-3593-3p	miR-27b-3p		
				miR-149-3p		
				miR-150-3p		
				miR-92b		
				miR-133c		
				miR-139-3p		
				miR-290		

These miRNA signatures were derived from previously published miRNA profiling data from primary rat hepatic stellate cells activated in culture (21), rat liver sinusoidal endothelial cells (LSECs) compared with other liver cell types (22), and mouse Kupffer cells (KCs) at 3 days after 70% partial hepatectomy (PHx) (23). The subset of miRNAs overlapping with the present data set is indicated.

### Manipulation of miR-21 Levels In Vitro Supports Evidence of Stellate Cell Mirna Signature in Whole Tissue

**Figure 5.**
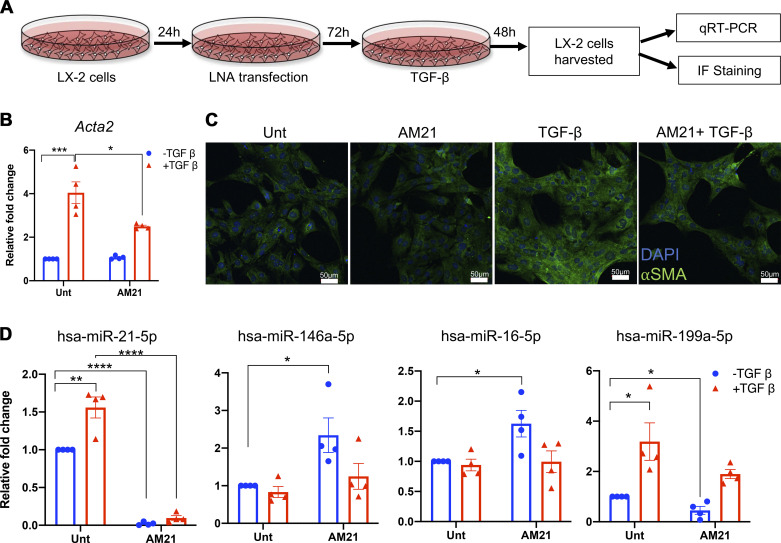
In vitro manipulation of miR-21 suggests a stellate cell-specific miRNA signature in whole tissue. *A*: schematic representation of locked nucleic acid (LNA) transfection and TGF-β stimulation in human hepatic stellate cell line. LX-2 cells were seeded for 24 h to ensure adherence, then simultaneously serum-starved and transfected for 72 h with 10 nM of LNA inhibitor targeting miR-21. Cells were then stimulated with 5 ng/mL of recombinant TGF-β for 48 h then harvested for RNA isolation. *Acta2* levels measured by qRT-PCR (*B*) and qualitative immunofluorescent imaging (*C*) provide evidence of stellate cell activation by TGF-β. *D*: qRT-PCR analysis of miRNAs from LX-2 cells transfected with anti-miR-21 (AM21) and stimulated with TGF-β. Values are given as means ± SE (*n* = 4); ****P < 0.0001, ***P < 0.001, **P < 0.01, and *P < 0.05. The relative fold change represented is the fold change versus untreated sample.

To validate our GSEA results indicating HSC differential miRNA signatures, we used LX2 cells, an immortalized human stellate cell line (Fig. 5*A*) (28). LX-2 cells were transfected using a locked nucleic acid-based approach to inhibit miR-21 and cells were stimulated with TGF-β. We first validated in vitro activation of LX-2 cells using *Acta2* expression as a marker. Treatment with TGF-β was sufficient to induce *Acta2* upregulation at both mRNA (Fig. 5*B*) and protein levels (Fig. 5*C*). A subset of miRNAs was selected based on our Nanostring data and used for GSEA (21). As observed in whole tissue samples, miR-21 and miR-146a displayed an anticorrelative relationship, with miR-146a increasing significantly in response to AM21 treatment (Fig. 5*D*). Treatment with TGF-β, which promotes activation in stellate cells, causes a decrease in miR-146a levels in conjunction with AM21 similar to the changes seen in vivo after PHx. miR-16, which has previously been shown to decrease in response to stellate cell activation (21), did not show a significant change with TGF-β alone, but AM21 treatment was sufficient to significantly increase miR-16 expression (Fig. 5*D*). Interestingly, the combination of TGF-β and AM21 returned miR-16 to baseline expression levels, supporting the idea that miR-16 expression is decreased in the hepatic stellate cell activation response. Lastly, miR-199a has been reported (21) to increase in activated HSCs, and we observed a similar result in response to TGF-β treatment (Fig. 5*D*). Treatment with AM21 led to a decrease in miR-199a expression and abrogated the increase in expression seen with TGF-β alone. Taken together, our findings show that these miRNA profiles, observed in whole tissue, are supportive of the major hepatic stellate cell role in the liver regenerative response, particularly in attenuating the response in the chronic ethanol group, as well as mediating the recovery of regeneration response following miR-21 inhibition.

## DISCUSSION

This study further explores the relationship between miRNA expression and liver regeneration in the context of chronic alcohol intake. We have shown that prolonged intake of ethanol in rats leads to dysregulation of normal miRNA expression patterns, which are further altered by additional insult, here in the form of a 70% partial hepatectomy. We have also shown that changes in miRNA expression patterns show altered dynamics between control and ethanol-fed animals. Furthermore, using a novel technique for exhaustive and unbiased comparative data analysis, we quantitatively identified clusters of miRNAs whose expression significantly changed during liver regeneration, between control and ethanol-fed animals. One of these miRNAs in particular, miR-21, plays an important role in liver regeneration and is significantly upregulated by chronic ethanol consumption (9, 24, 29). Preliminary analysis using a small and targeted cohort of miRNAs showed some changes in response to miR-21 inhibition but was limited in scope (9). The use of Nanostring nCounter assays allowed us an unbiased view of global miRNA expression in whole liver tissue. Using a miR21-specific LNA oligonucleotide inhibitor, we showed that several previously unremarked miRNAs display altered expression patterns in response to knockdown of miR-21 as well as by ethanol consumption during liver regeneration. Furthermore, gene ontology analysis was used to identify mechanisms by which these miRNAs may regulate liver regeneration, with the findings consistent with previous reports on tissue repair and particularly HSC function.

Although this study focused on miR-21, there are many miRNAs of interest in the context of liver regeneration, particularly those whose expression is correlated or anticorrelated with miR-21. For instance, miR-146a, which we identified as being negatively correlated with miR-21, is known to play an important role in regulating hepatic stellate cell activation, whose functional role in liver regeneration has been widely studied (4, 21, 25). We have also identified several other miRNAs that display similar changes in expression patterns as those seen in miR-21, such as miR-340-5p, which represent possible targets for manipulation in future experiments.

In addition, gene set enrichment analysis shows that global miRNA expression is potentially reflective of changes occurring in nonparenchymal cells, particularly HSCs. A data set from a previously published paper was used for this analysis—examining miRNA changes in activated versus quiescent rat primary stellate cells (21). By comparing this set of miRNAs with our observed data, we see that there is an outsized influence of this nonparenchymal cell type compared with its relative abundance in total liver. Stellate cell-relevant miRNA expression patterns in ethanol-fed animals reflect activated states in these cell types at both baseline and during regeneration, whereas pair-fed animals only display such changes in response to liver injury. Persistent activation of hepatic stellate cells has been linked to development of liver fibrosis and impaired regeneration (30), suggesting that the heightened stellate cell activity induced by ethanol adaptation may be leading to improper liver response to injury. Although similar analyses of LSEC and KC data sets did not show enrichment of the corresponding miRNA signatures in the present data set based on whole tissue samples, their potential contributions to the liver regeneration and ethanol-mediated disruption of response to partial hepatectomy should not be discounted. Follow-on studies focused on the role of individual nonparenchymal cell types in mechanisms relevant to alcohol-associated liver disease could be pursued using transcriptomics and small RNA profiling, ideally at the single-cell resolution, as has been recently demonstrated in the liver tissue (31–33). In addition, in situ hybridization approaches can be informative in localizing the expression and changes in miRNA levels to specific liver cell types. We note that several miRNAs in the nonparenchymal cell associated signatures (particularly for HSCs and KCs) are not specific to those cell types, even as the pattern of change in miRNA expression may be particular to those cells. Hence, analysis of the results from in situ hybridization and single-cell profiling methods needs to account for tissue-wide expression and probe for differential regulation likely to occur in a cell-type specific manner.

Gene ontology analysis of putative miRNA targets reveals several critical functions, including regulation of cell death, differentiation, and development, may be shifted by differential miRNA expression (Table 1). In particular, changes in the TGF-β signaling pathway have been widely studied in the context of liver injury and repair, and seem to be directly regulated by several miRNAs, including miR-21 and miR-146a (26). Beyond the TGF-β pathway, these two miRNAs have been implicated in additional pathways and disease states including several types of cancer and control regulation of protein phosphatase activity, matrix remodeling, and cytokine signaling, among others. In addition to controlling the levels of targeted genes directly, there is evidence for miRNA-miRNA regulation through downstream effects. miR-21 and miR-146a displayed pronounced anticorrelative expression patterns in both whole tissue and cell isolates. In addition, miRs-16 and 199a showed differential expression in human stellate cells in response to either TGF-β or AM21, or both. These findings clearly show that although miR-21 is not a master regulator of stellate cell miRNA networks, it plays a critical role in regulating the activation response and ultimately contributes to overall liver injury response. Taken together, these results reveal the complexity underlying the regulation of liver regeneration and demonstrate the need for further study with network analysis, computational modeling, and experimental validation.

Our results suggest that the alteration of nonparenchymal cell activity, in particular hepatic stellate cell function, and corresponding loss of regenerative ability in alcohol-fed livers is due to a shift in miRNA expression levels, fundamentally changing key gene regulatory networks involved in the repair and regeneration response. Our analysis has shown dramatic shifts in miRNA expression levels between ethanol-adapted and pair-fed animals during liver regeneration, which can be further altered by inhibition of a single miRNA, miR-21. Gene set enrichment analysis using curated sets of miRNAs shows a distinct, specific signature of hepatic stellate cells miRNAs within whole tissue data. These changes have been borne out in the LX-2 primary human hepatic stellate cell line as well. The dynamics between miR-21 and miR-146a have been shown to play critical roles in the regulation of HSC-specific signaling networks, further supporting a potential mechanism of action for the impairment of liver regeneration (26). In summary, our results have identified novel targets for the study of alcohol-associated liver disease and the role therein of miRNA regulatory networks, which can be further explored using in vitro models of HSC activation as well as in vivo models of chronic ethanol consumption using manipulations of the key miRNAs identified in the present study.

## DATA AVAILABILITY

The microRNA profiling data that support this study are available at NCBI Gene Expression Omnibus: GEO accession ID GSE171438 (https://www.ncbi.nlm.nih.gov/geo/query/acc.cgi?acc=GSE171438).

## GRANTS

This research was supported by National Institutes of Health National Institute on Alcohol Abuse and Alcoholism Grants R01 AA018873 (to R. Vadigepalli and J. B. Hoek), F31 AA024969 (to A. Parrish), and T32 AA007463 (to J. B. Hoek), and National Institute of Biomedical Imaging and Bioengineering Grant U01 EB023224 (to R. Vadigepalli and J. B. Hoek).

## DISCLOSURES

No conflicts of interest, financial or otherwise, are declared by the authors.

## AUTHOR CONTRIBUTIONS

J.B.H. and R.V. conceived and designed research; A.P., A.S., and E.J. performed experiments; A.P., A.S., and R.V. analyzed data; A.P., A.S., J.B.H., and R.V. interpreted results of experiments; A.P. and A.S. prepared figures; A.P. and A.S. drafted manuscript; J.B.H. and R.V. edited and revised manuscript; J.B.H. and R.V. approved final version of manuscript.

## References

[1] Altamirano J, Qi Q, Choudhry S, Abdallah M, Singal AK, Humar A, Bataller R, Borhani AA, Duarte-Rojo A. Non-invasive diagnosis: non-alcoholic fatty liver disease and alcoholic liver disease. Transl Gastroenterol Hepatol 5: 31, 2020. doi:10.21037/tgh.2019.11.14. 32258535 PMC7063491

[2] Farooq MO, Bataller R. Pathogenesis and management of alcoholic liver disease. Dig Dis 34: 347–355, 2016. doi:10.1159/000444545. 27170388 PMC4910523

[3] Achanta S, Verma A, Srivastava A, Nilakantan H, Hoek JB, Vadigepalli R. Single-cell gene expression analysis identifies chronic alcohol-mediated shift in hepatocyte molecular states after partial hepatectomy. Gene Expr 19: 97–119, 2019. doi:10.3727/105221618X15361728786767. 30189915 PMC6466177

[4] Kuttippurathu L, Juskeviciute E, Dippold RP, Hoek JB, Vadigepalli R. A novel comparative pattern analysis approach identifies chronic alcohol mediated dysregulation of transcriptomic dynamics during liver regeneration. BMC Genomics 17: 260, 2016. doi:10.1186/s12864-016-2492-x. 27012785 PMC4807561

[5] Bala S, Csak T, Kodys K, Catalano D, Ambade A, Furi I, Lowe P, Cho Y, Iracheta-Vellve A, Szabo G. Alcohol-induced miR-155 and HDAC11 inhibit negative regulators of the TLR4 pathway and lead to increased LPS responsiveness of Kupffer cells in alcoholic liver disease. J Leukoc Biol 102: 487–498, 2017. doi:10.1189/jlb.3A0716-310R. 28584078 PMC6608073

[6] Kerr TA, Korenblat KM, Davidson NO. MicroRNAs and liver disease. Transl Res 157: 241–252, 2011. doi:10.1016/j.trsl.2011.01.008. 21420035 PMC3063952

[7] Wang XW, Heegaard NH, Orum H. MicroRNAs in liver disease. Gastroenterology 142: 1431–1443, 2012. doi:10.1053/j.gastro.2012.04.007. 22504185 PMC6311104

[8] Francis H, McDaniel K, Han Y, Liu X, Kennedy L, Yang F, McCarra J, Zhou T, Glaser S, Venter J, Huang L, Levine P, Lai JM, Liu CG, Alpini G, Meng F. Regulation of the extrinsic apoptotic pathway by microRNA-21 in alcoholic liver injury. J Biol Chem 289: 27526–27539, 2014. doi:10.1074/jbc.M114.602383. 25118289 PMC4183793

[9] Juskeviciute E, Dippold RP, Antony AN, Swarup A, Vadigepalli R, Hoek JB. Inhibition of miR-21 rescues liver regeneration after partial hepatectomy in ethanol-fed rats. Am J Physiol Gastrointest Liver Physiol 311: G794–G806, 2016. doi:10.1152/ajpgi.00292.2016. 27634014 PMC5130549

[10] Karakatsanis A, Papaconstantinou I, Gazouli M, Lyberopoulou A, Polymeneas G, Voros D. Expression of microRNAs, miR-21, miR-31, miR-122, miR-145, miR-146a, miR-200c, miR-221, miR-222, and miR-223 in patients with hepatocellular carcinoma or intrahepatic cholangiocarcinoma and its prognostic significance. Mol Carcinog 52: 297–303, 2013. doi:10.1002/mc.21864. 22213236

[11] Marquez RT, Wendlandt E, Galle CS, Keck K, McCaffrey AP. MicroRNA-21 is upregulated during the proliferative phase of liver regeneration, targets Pellino-1, and inhibits NF-kappaB signaling. Am J Physiol Gastrointest Liver Physiol 298: G535–G541, 2010. doi:10.1152/ajpgi.00338.2009. 20167875 PMC2853303

[12] Ng R, Song G, Roll GR, Frandsen NM, Willenbring H. A microRNA-21 surge facilitates rapid cyclin D1 translation and cell cycle progression in mouse liver regeneration. J Clin Invest 122: 1097–1108, 2012. doi:10.1172/JCI46039. 22326957 PMC3287214

[13] Lieber CS, DeCarli LM. The feeding of alcohol in liquid diets: two decades of applications and 1982 update. Alcohol Clin Exp Res 6: 523–531, 1982. doi:10.1111/j.1530-0277.1982.tb05017.x. 6758624

[14] Robinson MD, McCarthy DJ, Smyth GK. edgeR: a bioconductor package for differential expression analysis of digital gene expression data. Bioinformatics 26: 139–140, 2010. doi:10.1093/bioinformatics/btp616. 19910308 PMC2796818

[15] Law CW, Chen Y, Shi W, Smyth GK. voom: precision weights unlock linear model analysis tools for RNA-seq read counts. Genome Biol 15: R29, 2014. doi:10.1186/gb-2014-15-2-r29. 24485249 PMC4053721

[16] Ritchie ME, Phipson B, Wu D, Hu Y, Law CW, Shi W, Smyth GK. limma powers differential expression analyses for RNA-sequencing and microarray studies. Nucleic Acids Res 43: e47, 2015. doi:10.1093/nar/gkv007. 25605792 PMC4402510

[17] Phipson B, Lee S, Majewski IJ, Alexander WS, Smyth GK. Robust hyperparameter estimation protects against hypervariable genes and improves power to detect differential expression. Ann Appl Stat 10: 946–963, 2016. doi:10.1214/16-AOAS920. 28367255 PMC5373812

[18] Götze S, Schumacher EC, Kordes C, Häussinger D. Epigenetic changes during hepatic stellate cell activation. PLoS One 10: e0128745, 2015. doi:10.1371/journal.pone.0128745. 26065684 PMC4466775

[19] Betel D, Wilson M, Gabow A, Marks DS, Sander C. The microRNA.org resource: targets and expression. Nucleic Acids Res 36: D149–153, 2008. doi:10.1093/nar/gkm995. 18158296 PMC2238905

[20] Huang da W, Sherman BT, Lempicki RA. Systematic and integrative analysis of large gene lists using DAVID bioinformatics resources. Nat Protoc 4: 44–57, 2009. doi:10.1038/nprot.2008.211. 19131956

[21] Maubach G, Lim MC, Chen J, Yang H, Zhuo L. miRNA studies in in vitro and in vivo activated hepatic stellate cells. World J Gastroenterol 17: 2748–2773, 2011. doi:10.3748/wjg.v17.i22. 21734783 PMC3122263

[22] Oda S, Takeuchi M, Akai S, Shirai Y, Tsuneyama K, Yokoi T. miRNA in rat liver sinusoidal endothelial cells and hepatocytes and application to circulating biomarkers that discern pathogenesis of liver injuries. Am J Pathol 188: 916–928, 2018. doi:10.1016/j.ajpath.2017.12.007. 29353062

[23] Elchaninov A, Nikitina M, Vishnyakova P, Lokhonina A, Makarov A, Sukhikh G, Fatkhudinov T. Macro- and microtranscriptomic evidence of the monocyte recruitment to regenerating liver after partial hepatectomy in mouse model. Biomed Pharmacother 138: 111516, 2021. doi:10.1016/j.biopha.2021.111516. 33765583

[24] Dippold RP, Vadigepalli R, Gonye GE, Hoek JB. Chronic ethanol feeding enhances miR-21 induction during liver regeneration while inhibiting proliferation in rats. Am J Physiol Gastrointest Liver Physiol 303: G733–G743, 2012. doi:10.1152/ajpgi.00019.2012. 22790595 PMC3468539

[25] He Y, Huang C, Sun X, Long XR, Lv XW, Li J. MicroRNA-146a modulates TGF-β1-induced hepatic stellate cell proliferation by targeting SMAD4. Cell Signal 24: 1923–1930, 2012. doi:10.1016/j.cellsig.2012.06.003. 22735812

[26] Kuttippurathu L, Parrish A, Vadigepalli R. Integrated computational model of intracellular signaling and microrna regulation predicts the network balances and timing constraints critical to the hepatic stellate cell activation process. Processes 2: 773–794, 2014. doi:10.3390/pr2040773.

[27] Zhang Z, Zhang Y, Sun XX, Ma X, Chen ZN. microRNA-146a inhibits cancer metastasis by downregulating VEGF through dual pathways in hepatocellular carcinoma. Mol Cancer 14: 5, 2015. doi:10.1186/1476-4598-14-5. 25608619 PMC4326400

[28] Xu L, Hui AY, Albanis E, Arthur MJ, O'Byrne SM, Blaner WS, Mukherjee P, Friedman SL, Eng FJ. Human hepatic stellate cell lines, LX-1 and LX-2: new tools for analysis of hepatic fibrosis. Gut 54: 142–151, 2005. doi:10.1136/gut.2004.042127. 15591520 PMC1774377

[29] Dippold RP, Vadigepalli R, Gonye GE, Patra B, Hoek JB. Chronic ethanol feeding alters miRNA expression dynamics during liver regeneration. Alcohol Clin Exp Res 37, Suppl 1: E59–E69, 2013. doi:10.1111/j.1530-0277.2012.01852.x. 22823254 PMC3482264

[30] Reeves HL, Friedman SL. Activation of hepatic stellate cells—a key issue in liver fibrosis. Front Biosci 7: d808–d826, 2002. doi:10.2741/reeves. 11897564

[31] MacParland SA, Liu JC, Ma XZ, Innes BT, Bartczak AM, Gage BK, et al. Single cell RNA sequencing of human liver reveals distinct intrahepatic macrophage populations. Nat Commun 9: 4383, 2018. doi:10.1038/s41467-018-06318-7. 30348985 PMC6197289

[32] Sabater L, Locatelli L, Oakley F, Hardy T, French J, Robinson SM, Sen G, Mann DA, Mann J. RNA sequencing reveals changes in the microRNAome of transdifferentiating hepatic stellate cells that are conserved between human and rat. Sci Rep 10: 21708, 2020. doi:10.1038/s41598-020-78776-3. 33303921 PMC7728773

[33] Walesky CM, Kolb KE, Winston CL, Henderson J, Kruft B, Fleming I, Ko S, Monga SP, Mueller F, Apte U, Shalek AK, Goessling W. Functional compensation precedes recovery of tissue mass following acute liver injury. Nat Commun 11: 5785, 2020. doi:10.1038/s41467-020-19558-3. 33214549 PMC7677389

